# Effect of rosuvastatin on hyperuricemic rats and the protective effect on endothelial dysfunction

**DOI:** 10.3892/etm.2014.2027

**Published:** 2014-10-15

**Authors:** DILIDAER XILIFU, ABULIZI ABUDULA, NIJIATI REHEMU, LONG ZHAO, XINRONG ZHOU, XIANGYANG ZHANG

**Affiliations:** 1 Department of Cardiology, The First Affiliated Hospital, Xinjiang Medical University, Ürümqi, Xinjiang 830011, P.R. China; 2 Xinjiang Key Laboratory of Molecular Biology and Endemic Diseases, Xinjiang Medical University, Ürümqi, Xinjiang 830011, P.R. China; 3 Department of Pathology, School of Basic Medicine, Xinjiang Medical University, Ürümqi, Xinjiang 830011, P.R. China

**Keywords:** hyperuricemia, uric acid, rosuvastatin, endothelial dysfunction, nitric oxide

## Abstract

Endothelial dysfunction plays a key role in the development of cardiovascular diseases, renal injuries and hypertension induced by hyperuricemia. Therapies targeting uric acid (UA) may be beneficial in cardiovascular diseases. In the present study, the effect of rosuvastatin, a 3-hydroxy-3-methylglutaryl coenzyme A reductase inhibitor, was investigated to determine whether rosuvastatin improves endothelial dysfunction via the endothelial nitric oxide (NO) pathway and delays the pathogenesis of endothelial dysfunction in hyperuricemic rats. A total of 72 Sprague-Dawley rats (age, 8 weeks) were randomly divided into six groups (12 rats per group), including the control, model, 2.5 mg/kg/day rosuvastatin, 5 mg/kg/day rosuvastatin, 10 mg/kg/day rosuvastatin and 53.57 mg/kg/day allopurinol groups. The model, rosuvastatin and allopurinol rats were subjected to hyperuricemia, induced by the administration of yeast extract powder (21 g/kg/day) and oxonic acid potassium salt (200 mg/kg/day). The hyperuricemic rats were treated with 2.5, 5.0 or 10.0 mg/kg/day rosuvastatin orally for six weeks, while rats treated with allopurinol (53.57 mg/kg/day) were used as a positive control. The serum levels of NO and the gene expression levels of endothelial NO synthase in the aortic tissue increased, whereas the serum levels of UA, endothelin-1 and angiotensin II decreased in the hyperuricemic rats treated with rosuvastatin, particularly at a high rosuvastatin dose (10 mg/kg/day). In addition, the curative effect of the 10 mg/kg/day rosuvastatin group was evidently higher compared with the allopurinol group. Therefore, rosuvastatin may be a novel drug candidate for the treatment of hyperuricemia due to its endothelial protective properties.

## Introduction

Endothelial tissues are active and dynamic, lining the entire vascular system and controlling important functions (1). The endothelium serves as a barrier between the blood and tissues, actively participates in the regulation of vascular function and is critical to the biology of normal tissues. Tissue health is often synonymous with endothelial integrity. Vascular homeostasis and tone are controlled by the endothelium via the synthesis and release of a number of endothelium-derived relaxing and constricting substances (2), including nitric oxide (NO), angiotensin II (Ang II) and endothelin-1 (ET-1) (3,4). Quiescent endothelial cells suppress all phases of vascular diseases, including the degree of injury, local thrombosis, inflammation, proliferation and matrix remodeling, while an injured or dysfunctional endothelium can promote such events (5). Impaired NO bioavailability represents the central feature of endothelial dysfunction, which is a common abnormality occurring in a number of vascular diseases (6).

Hyperuricemia is a complex metabolic disease that can develop into gout (7). An increased serum level of uric acid (UA) is hypothesized to be independently associated with an increased risk of mortality from cardiovascular diseases (8). The serum levels of UA increase physiologically and gradually with aging due to the excess dietary purine or ethanol intake (9,10).

Increasing evidence indicates that hyperuricemia is associated with endothelial dysfunction (11,12). The occurrence of abnormal endothelial function in individuals suffering with hyperuricemia is a cause of health problems, since endothelial dysregulation is a major determinant of atherosclerosis in the early pathophysiological stages, and has been demonstrated to occur in a time-dependent manner in patients with coronary artery disease, hypertension and type 2 diabetes (13,14). Thus, strategies for improving endothelial function are currently under investigation.

Statins are 3-hydroxy-3-methylglutaryl coenzyme A (HMG-CoA) reductase (HMGR) inhibitors, administered to inhibit cholesterol synthesis, thereby lowering the serum levels of cholesterol (15). In addition to lowering the levels of low-density lipoproteins, statins exhibit pleiotropic effects, such as improving endothelial function, inhibiting the synthesis of endothelins, reducing coronary artery thrombus formation and inhibiting the proliferation of smooth muscle cells, as well as anti-inflammatory, antioxidant and antiplatelet effects (16). Endothelial function is critical for cardiovascular function, and serves as a surrogate marker in monitoring the efficacy of statin treatment, which indicates novel, potential applications for these drugs in the primary and secondary prevention of acute cardiovascular events (17).

Among the synthetic, type 2 or second-generation statins, rosuvastatin is a sulphur-containing hydrophilic statin with multiple binding sites that forms a strong interaction with HMGR. Thus, rosuvastatin provides more potent enzyme inhibition compared with other statins. Rosuvastatin, similar to other statins, is a competitive antagonist of HMGR, competing directly with the endogenous substrate for the active site cavity of the enzyme. The drug has an affinity for the HMGR active site that is >10^4^-fold higher than the affinity for HMG-CoA (18,19).

In the present study, yeast extract powder (YEP) and oxonic acid (OA) potassium salt were used as revulsants to establish a hyperuricemic rat model. Interventions with various rosuvastatin calcium dosages were applied to analyze the effect of rosuvastatin on the experimental animal model. In the present study, a hyperuricemic rat model was established by intragastrical feeding of yeast extract in combination with various doses of OA potassium salt through intraperitoneal injection. The model was treated with various rosuvastatin doses. By determining the drug effect on various factors associated with the vascular endothelial function of the rats, the present study may provide important evidence on the application of rosuvastatin in the clinical treatment of hyperuricemia.

## Materials and methods

### Animals

A total of 72 male Sprague-Dawley rats (age, 8 weeks; weight, 203.8±32.15 g) were provided by the Experimental Animal Center of Xinjiang Medical University (Ürümqi, China). The rats were fed a standard laboratory diet, available at the center, prior to the initiation of the study. The body weight of the rats was measured twice a week in order to adjust the drug dosage to the rat weight. The study was conducted in strict accordance with the recommendations in the Guide for the Care and Use of Laboratory Animals of the National Institutes of Health (20). The animal use protocol was reviewed and approved by the Ethical Committee and Institutional Animal Care and Use Committee of the First Affiliated Hospital of Xinjiang Medical University (approval no. 20100310003; Ürümqi, China).

### Establishment of an animal model and drug intervention

An experimental animal model of hyperuricemia was established according to the reference data found in previous studies (21). In the present study, animal modeling and drug intervention were initiated simultaneously after randomly dividing the 72 male rats into six groups (12 rats per group); the drug intervention was continued for six weeks. To establish the animal model, the rats were intragastrically administered 21 g/kg/day YEP (production batch no. 20090705; Beijing Aoboxing Biological Technology Co., Ltd, Beijing, China) mixed with standard feed at a proportion of 1:4, and intraperitoneally injected with 200 mg/kg/day OA (production batch no. 20120312; Sigma-Aldrich Co., Munich, Germany). A stock solution of 5 mg/ml rosuvastatin (production batch no. EK188; AstraZeneca Pharmaceuticals Co., Södertälje, Sweden) was prepared for the drug intervention. The rats were administered 2.5, 5.0 and 10.0 mg/kg/day rosuvastatin by intravenous injection, while 53.57 mg/kg/day allopurinol (Beijing Double-Crane Pharmaceutical Co., Ltd, Beijing, China) was administered as the positive control.

### Specimen collection

Anticoagulated blood samples (2 ml) were collected by excising the eyeballs of the rats prior to the experiment (week 0) and at weeks 2, 4 and 6. Following separation through natural blood clotting and centrifugation (1509.3 × g for 15 min; Allegra 64R, Beckmann Coulter, Miami, FL, USA), the plasma was collected and stored at −80°C until required for analysis. Blood samples were collected in the morning following a minimum fasting period of 10 h. After six weeks, the rats were sacrificed using 40 mg/kg sodium pentobarbital. The entire thoracic aorta of each rat was removed, snap frozen in liquid nitrogen, fixed in 1–4% neutral-buffered formalin, embedded in paraffin and cut into 4–5-μm serial sections for immunohistochemical staining.

### Evaluated serum parameters

Lipids were extracted from 100-μl plasma samples and analyzed to obtain the serum levels of UA, NO, ET-1 and Ang II using commercially available ELISA detection kits (R&D Systems, Inc., Minneapolis, MN, USA) and a DxC800 Synchron Biochemical Analysis System (Beckman Coulter, Inc., Brea, CA, USA) according to the manufacturer’s instructions and standard clinical protocols.

### Immunofluorescence staining

Tissue specimens were cut into 4-μm sections, mounted on glass slides by heating at 56°C, dewaxed with standard xylene and rehydrated using graded alcohol solutions and water. The internal enzyme activity of the tissue sections was inhibited by H_2_O_2_ treatment. Standard immunohistochemical staining was performed using a rabbit antieNOS polyclonal primary antibody (Santa Cruz Biotechnology, Inc., Santa Cruz, CA, USA) followed by detection with a goat anti-rabbit polyclonal secondary antibody conjugated with horseradish peroxidase (Beijing Zhongshan, Beijing, China). Negative control sections of each specimen were processed using the same method, with omission of the primary antibodies.

The horseradish peroxidase antigen was positively stained with a yellow, claybank or brown color in the nucleus. The intensity and percentage of positive cells in the sections were determined by selecting five scopes at a high magnification (×400) under a light microscope (inverted fluorescence microscope; BA120, Motic, HongKong, China). The percentage of positive cells was scored as zero for 0–5%, one for 5–25%, two for 25–75% and three for 75–100% (21). The staining intensity was scored as zero, one, two or three, indicating the absence of staining, weak yellow, claybank and brown staining, respectively. The sum of the two scores was used to identify three categories of expression: Total loss (<1), partial loss (1–3) and normal (>4).

### Statistical analysis

Statistical analysis was performed using SPSS 17.0 software (SPSS, Inc., Chicago, IL, USA). Values are presented as the mean ± standard deviation. Statistical significance between two groups or among multiple groups was evaluated using one-way analysis of variance, followed by Fisher’s least significant difference or Tamhane’s post hoc tests to correct for multiple comparisons. P<0.05 was considered to indicate a statistically significant difference.

## Results

### Effects of rosuvastatin treatment on the serum UA level in hyperuricemic rats

In pilot experiments, the rats were treated with YEP by intragastrical feeding, in combination with various doses of OA by intraperitoneal injection, for six weeks. The optimal dose of OA for the establishment of the experimental rat model of hyperuricemia was found to be 200 mg/kg/day, which induced a significant increase in the serum level of UA, accompanied with evident morphological and pathological changes in the kidney, heart and arteries of the rats. Following intervention with various rosuvastatin doses, the drug was demonstrated to reduce or regulate the serum levels of UA in a dose- and time-dependent manner (Table I). The serum level of UA increased markedly in the hyperuricemic rats when compared with the normal animals after two weeks. However, no statistically significant differences were observed between the rosuvastatin-treated and untreated groups. After four weeks, the serum level of UA continued to increase in the model hyperuricemic rats when compared with the normal controls, indicating a time-dependent effect of YEP and OA in the hyperuricemic rat model. By contrast, a significant decrease was detected in the serum level of UA in the hyperuricemic rats treated with rosuvastatin, occurring in a dose- and time-dependent manner. The effect of rosuvastatin was found to be statistically significant after six weeks of treatment, since the serum UA level of rosuvastatin-treated groups was recovered to the level of normal animals or the allopurinol-treated group. The serum level of UA in the hyperuricemic rats treated with rosuvastatin recovered to that of the normal animals or the positive controls treated with allopurinol, particularly at a high rosuvastatin dose (10 mg/kg/day). When compared with the rosuvastatin-treated groups, the serum level of UA in the untreated hyperuricemic model rats continued to increase after six weeks. Thus, rosuvastatin may serve as a therapeutic agent against hyperuricemia, since the treatment was shown to reduce the serum level of UA in the hyperuricemic experimental rat model and produce an effect comparable with or higher than the effect of allopurinol.

### Effects of rosuvastatin treatment on the serum level of proteins associated with vascular endothelial function in hyperuricemic rats

Changes in the serum levels of ET-1, Ang II and NO reflect the function of vascular endothelial cells. An increase in the serum levels of ET-1 and Ang II, and a decrease in the serum level of NO, were detected following the establishment of the hyperuricemic rat model (Table II). Rosuvastatin treatment resulted in the recovery of the serum levels of ET-1, Ang II and NO induced by hyperuricemia. In addition, the effect was enhanced in a dose-dependent manner, exhibiting a statistically significant decrease in the serum levels of ET-1 and Ang II or an increase in the serum level of NO after four weeks of treatment, when compared with the model group. Recovery to normal levels occurred after six weeks of drug treatment and was clearly enhanced compared with the persistently increasing serum levels of the proteins in the model hyperuricemic rats, particularly at a high rosuvastatin dose (10 mg/kg/day). Treatment with allopurinol (positive control) also resulted in the recovery of the serum levels; however, the recovery was to a lower extent compared with that of rosuvastatin.

### Effects of rosuvastatin on the expression of endothelial NO synthase (eNOS) in the aortic tissues of hyperuricemic rats

**Figure 1:**
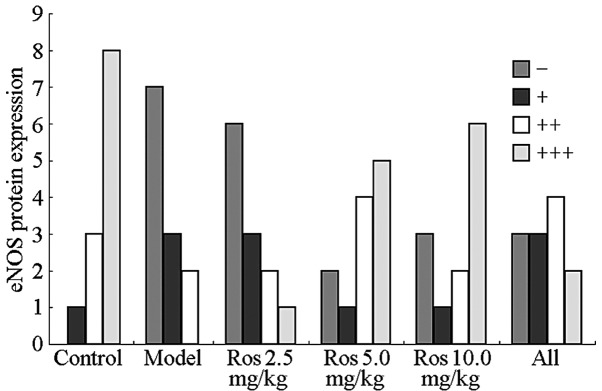
Determination of eNOS expression in the arterial tissue of rats in the different groups. Ros, rosuvastatin; eNOS, endothelial nitric oxide synthase.

Effects of rosuvastatin on the expression level of eNOS in the aortic tissues of the hyperuricemic rats are demonstrated in Fig. 1. Rosuvastatin was shown to affect the serum level of NO after two weeks of treatment, indicating that the serum level of NO was a fine-tuning factor in hyperuricemia and was more susceptible to treatment compared with the other factors analyzed. To determine the underlying mechanisms and confirm the aforementioned conclusion, the expression level of eNOS, a rate-limiting enzyme in NO metabolism, was determined in the aortic tissues of the rats by an immunohistochemical assay.

Protein expression of eNOS was observed primarily in the nucleus and partially in the cytoplasm of aortic tissue endothelial cells. Positive expression was observed as granules with colors ranging between yellow and brown, representing weak to strong protein expression. Protein expression of eNOS was strongly positive in the aortic specimens from the normal group, whereas the expression was negative in the hyperuricemic model group (Fig. 1), correlating with the alteration in the serum level of NO (Table II). After six weeks of rosuvastatin treatment, the expression level of eNOS in the aortic tissues of the hyperuricemic rats increased gradually in a dose-dependent manner, and became fully restored to normal levels when treated with a high dose. The effect of rosuvastatin in improving the endothelial function of the aorta in terms of eNOS expression was found to be significantly higher compared with the allopurinol-treated group.

## Discussion

In the present study, a hyperuricemic rat model was generated using YEP and potassium oxonate to investigate the pharmacological effect of rosuvastatin on the regulation of endothelial function in hyperuricemic rats. Endothelial function was assessed by analyzing the upregulation of UA, ET-1 and Ang II, and the downregulation of NO, as well as the inhibition of eNOS expression in aortic tissues.

The results of the present study support the hypothesis that UA is a significant factor in the pathogenesis of endothelial dysfunction, which is consistent with the results obtained by previous studies (22,23). A novel mechanism explaining this phenomenon was also proposed. Xanthine dehydrogenases degrade purine metabolic products, whereas xanthine oxidases are mainly released by vascular endothelial cells. UA is a xanthine oxidase, and when the level of UA is increased, xanthine oxidase-induced oxidative stress is enhanced, generating and sequentially activating further oxygen free radicals, including the endothelial superoxide anion, hydroxyl radical and hydrogen peroxide. The oxygen free radicals subsequently produce endoperoxide via the activation of epoxidase-1 and reactivate the tyrosine protein kinase receptor, leading to acetylcholine endothelium-dependent vasoconstriction, increased resistance, angiostatic disharmony and endothelial dysfunction (24). In the present study, a reduction in the levels of plasma nitrates and nitrites was observed in the hyperuricemic rats, which was consistent with the endothelial dysfunction and decreased NO production (25). UA can also increase renin expression *in vivo*, stimulate the production of Ang II and increase the expression of Ang II receptor type 1 in cultured vascular smooth muscle cells. An increase in the expression levels of UA can also increase the expression levels of ET-1 in cardiac fibroblasts and vascular smooth muscle cells (26). An *in vivo* study demonstrated that hyperuricemia may injure endothelial function via resistin-dependent mechanisms (14).

The results of the present study indicate that rosuvastatin may restore the expression levels of ET-1 and Ang II, which are closely associated with endothelial function, in hyperuricemic rats treated for two to six weeks in a dose- and time-dependent manner. Previous studies hypothesized that an interaction exists between ET-1 and Ang II, where ET-1 promotes the conversion of Ang I into Ang II by reducing the activity of the angiotensin-converting enzyme, whereas Ang II enhances the activity of the endothelin-converting enzyme (27,28). Statins may affect the endothelium through their antioxidant effects, attenuating the production of Ang II-induced free radicals in vascular smooth muscle cells by inhibiting the activity of Rac1-mediated NAD(P)H oxidase and downregulating the expression of the angiotensin receptor (29). In addition, a decreased production of ET-1 may inhibit the participation of the small G protein, RhoA (30). Takahashi *et al* demonstrated that statins prevent Ang II-induced vascular remodeling and oxidative stress, and suppress the Ang II-mediated activation of the extracellular signal-regulated kinase 1/2 in rat mesenteric arteries (31).

Experimental evidence has demonstrated that statins exhibit lipid-independent effects on endothelial function (32). Animal models have also revealed that statins can augment coronary blood flow, improve cardiac contractile function and inhibit leukocyte-endothelial cell interactions, primarily by enhancing the release of NO by endothelial cells, which is mediated by eNOS. An experimental study demonstrated the ability of statins to enhance the expression, increase the activity or prevent the inactivation of eNOS (33).

Previous study has shown that statins can increase the expression and activity of eNOS in vascular endothelial cells (34). In the present study, rosuvastatin was shown to increase the eNOS protein concentration in hyperuricemic rat endothelial cells, and prevent the hypoxia-dependent inhibition of eNOS synthesis. In addition, the eNOS protein concentration increased after six weeks of rosuvastatin treatment, particularly at a dosage of 10 mg/kg/day. These results indicate a protective effect of rosuvastatin on the synthesis of NO. Datar *et al* demonstrated that statins inhibit endothelial-dependent vascular relaxation in the aorta and increase the levels of reactive nitrogen and oxygen species (35). The translocation of eNOS to the intracellular structures may be responsible for the increased peroxynitrite formation. The authors hypothesized that lipophilic HMGR inhibitors may promote oxidative stress (36). Endothelium-derived NO is an important mediator of endothelial function, and several mechanisms have been reported for the upregulation of eNOS by statins (35). A possible mechanism involves the Rho/Rho-associated protein kinase signaling pathway, through which statins increase the stability of eNOS mRNA, leading to an increased expression of eNOS. A second important mechanism through which statins activate eNOS is via the serine-threonine protein kinase, Akt. Statins have been shown to rapidly promote the activation of Akt in endothelial cells, resulting in eNOS phosphorylation and increased angiogenesis, which is mediated by the tyrosine phosphorylation of heat shock protein 90 that activates eNOS (37). A third mechanism reported hypothesizes that statins regulate the activity of eNOS via their effects on caveolin-1. Caveolin-1 is an integral membrane protein that binds to eNOS in caveolae, thereby directly inhibiting the production of NO (38). The function of allopurinol, a xanthine oxidase inhibitor, in the prevention and treatment of hyperuricemia was limited due to the inhibitory effect of allopurinol on the biosynthesis of UA, evident only when high levels of UA accumulated in the blood circulation (39). In the present study, allopurinol exhibited positive effects on endothelial function; however, rosuvastatin was found to be more effective when compared with allopurinol.

In conclusion, rosuvastatin was demonstrated to reduce the serum level of UA and improve endothelial function in hyperuricemic rats. Hyperuricemia was mainly associated with an increase in the serum level of NO and reductions in the serum levels of ET-1 and Ang II. The present study provides important evidence on the clinical treatment of hyperuricemic patients with rosuvastatin. However, further studies are required to determine the mechanisms by which rosuvastatin reduces the serum level of UA and improves endothelial function.

## Figures and Tables

**Table I:** Changes in the levels of uric acid in the rats of the different groups (mean ± SD).

Group	YEP + OA	Rosuvastatin (mg/kg/day)	Allopurinol (mg/kg/day)	Uric acid (μmol/l)			

				Week 0	Week 2	Week 4	Week 6
Blank control	-	-	-	43.99±0.59	49.50±2.64^c^^e^^g^^i^^k^	45.31±1.66^c^^d^^g^^i^	45.14±0.89^c^^f^^i^
Model	✓	-	-	46.71±3.88	248.00±8.18^b^^e^^g^^i^^k^	352.25±5.35^b^^e^^g^^i^^k^	216.00±6.15^b^^e^^g^^i^^k^
Rosuvastatin	✓	2.50	-	43.29±0.90	124.75±16.79^b^^c^	118.50±4.65^b^^e^^g^^i^^k^	89.75±2.04^b^^c^^e^^g^^k^
	✓	5.00	-	44.63±1.09	129.00±5.05^b^^c^	100.50±1.72^b^^e^^g^^i^^k^	62.25±6.20^a^^c^^d^^h^^j^
	✓	10.00	-	42.28±0.61	112.75±13.89^b^^c^	67.00±10.27^a^^c^^e^	41.75±4.76^c^^f^^i^
Allopurinol	✓	-	53.75	40.69±5.51	103.75±10.03^b^^c^	59.25±2.86^a^^c^^e^	44.75±4.32^c^^f^^i^

a P<0.05 and

b P<0.01, vs. blank control group;

c P<0.01, vs. model group;

d P<0.05 and

e P<0.01, vs. 10 mg/kg/day rosuvastatin group;

f P<0.05 and

g P<0.01, vs. 5 mg/kg/day rosuvastatin group;

h P<0.05 and

i P<0.01, vs. 2.5 mg/kg/day rosuvastatin group;

j P<0.05 and

k P<0.01, vs. allopurinol group.

YEP, yeast extract powder; OA, oxonic acid.

**Table II:** Changes in the levels of endothelin-1, angiotensin II and nitric oxide in the rats of the different groups (mean ± SD).

Indicator	Blank control	Model (YEP+OA)	Rosuvastatin (YEP+OA)			Allopurinol (YEP+OA) 53.75 mg/kg/day

			2.5 mg/kg/day	5 mg/kg/day	10 mg/kg/day	
Endothelin-1 (μmol/l)						
Week 0	80.66±9.36	89.63±4.59	94.20±7.35	88.12±6.33	96.22±3.21	99.23±1.68
Week 2	87.45±6.53^b^^c^^e^^f^^g^	127.37±10.36^a^^e^	120.15±8.12^a^^g^	124.93±4.09^a^^g^	125.04±5.46^a^^g^	138.55±3.30^a^^c^^e^^f^
Week 4	94.12±5.47^b^^f^^g^	139.36±9.59^a^^c^	137.07±6.45^a^^c^	121.14±12.47^a^	110.70±9.22^b^^f^	126.29±13.21^a^
Week 6	85.37±3.85^b^^e^^f^^g^	163.28±7.50^a^^e^^f^^g^	112.95±10.83^a^^b^^c^	99.03±14.49^a^^b^^c^	87.59±9.02^b^^e^^f^^g^	96.62±8.12^a^^b^^c^
Angiotensin II (ng/l)						
Week 0	221.96±39.22	219.49±45.61	223.20±29.39	247.23±26.91	200.50±4.46	239.13±16.85
Week 2	231.43±41.99^b^^c^^e^^f^^g^	330.68±30.66^a^	342.80±24.33^a^	362.85±38.23^a^	333.56±49.22^a^	372.37±11.09^a^
Week 4	256.76±51.03^b^^f^^g^	403.26±69.57^a^^c^^e^^f^^g^	303.20±7.67^a^^b^	305.00±30.34^b^	274.22±48.68^b^	323.26±25.96^a^^b^
Week 6	219.56±13.25^b^^g^	419.69±50.99^a^^c^^e^^f^^g^	282.78±52.35^b^	250.07±64.71^b^	201.46±54.84^b^^g^	307.59±38.33^a^^b^^c^
Nitric oxide (mmol/l)						
Week 0	21.36±2.67	25.32±2.19	22.39±3.56	20.98±3.94	22.22±3.31	23.96±1.38
Week 2	25.21±2.90^b^^c^^d^	23.06±2.59^c^^e^	25.01±2.75^c^^d^	32.37±2.74^a^^b^^f^^g^	33.87±5.29^a^^b^^f^^g^	25.23±1.38^b^^c^^e^
Week 4	25.98±0.37^d^	19.56±4.34^c^^g^	25.60±1.31^b^^c^	28.66±1.79	35.79±0.61^a^^b^^f^^g^	25.95±2.78^c^
Week 6	24.55±1.99^b^	17.07±3.37^a^^c^	21.95±1.88	26.35±3.53^b^	28.32±8.71^b^	20.63±1.45^c^

a P<0.01, vs. control group;

b P<0.01, vs. model group;

c P<0.01, vs. 10 mg/kg/day rosuvastatin group;

d P<0.05 and

e P<0.01, vs. 5 mg/kg/day rosuvastatin group;

f P<0.01, vs. 2.5 mg/kg/day rosuvastatin group;

g P<0.01, vs. allopurinol group.

YEP, yeast extract powder; OA, oxonic acid.
